# Tisagenlecleucel vs. historical standard of care in children and young adult patients with relapsed/refractory B-cell precursor acute lymphoblastic leukemia

**DOI:** 10.1038/s41375-023-02042-4

**Published:** 2023-10-25

**Authors:** Arend v. Stackelberg, Katja Jäschke, Etienne Jousseaume, Corinna Templin, Ulli Jeratsch, Daniela Kosmides, Ingo Steffen, Nicola Gökbuget, Christina Peters

**Affiliations:** 1 Charité University Hospital Berlin, Berlin, Germany; 2 Novartis Pharma GmbH, Nürnberg, Germany; 3 Novartis Pharma AG, Basel, Switzerland; 4 Goethe University, Frankfurt, Germany; 5 St. Anna Children’s Hospital, Children’s Cancer Research Institute, University Vienna, Vienna, Austria

**Keywords:** Drug development, Acute lymphocytic leukaemia, Immunotherapy

## Abstract

In the absence of randomized controlled trials comparing tisagenlecleucel vs. standard of care (SOC) in pediatric and young adult patients with relapsed or refractory acute lymphoblastic leukemia (r/r ALL), the objective was to compare the efficacy of tisagenlecleucel with historical controls from multiple disease registries using patient-level adjustment of the historical controls. The analysis is based on patient-level data of three tisagenlecleucel studies (ELIANA, ENSIGN and CCTL019B2001X) vs. three registries in Germany/Austria. Statistical analyses were fully pre-specified and propensity score weighting of the historical controls by fine stratification weights was used to adjust for relevant confounders identified by systematic literature review. Results showed high comparability of cohorts after adjustment with absolute SMD ≤ 0.1 for all pre-specified confounders and favorable outcomes for tisagenlecleucel compared to SOC for all examined endpoints. Hazard ratios for OS_(Intention to treat)ITT,adjusted_, EFS_(Full analysis set)FAS,naïve_ and RFS_FAS,naïve_ were 0.54 (95% CI: 0.41–0.71, *p* < 0.001), 0.67 (0.52–0.86, *p* = 0.001) and 0.77 (0.51–1.18, *p* = 0.233). The OS_ITT, adjusted_, EFS_FAS,naïve_ and RFS_FAS,naive_ survival probability at 2 years was 59.49% for tisagenlecleucel vs. 36.16% for SOC population, 42.31% vs. 30.23% and 59.60% vs. 54.57%, respectively. Odds ratio for ORR_ITT,adjusted_ was 1.99 (1.33–2.97, *p* < 0.001). Results for OS and ORR were statistically significant after adjustment for confounders and provide evidence supporting a superiority of tisagenlecleucel in r/r ALL given the good comparability of cohorts after adjustment for confounders.

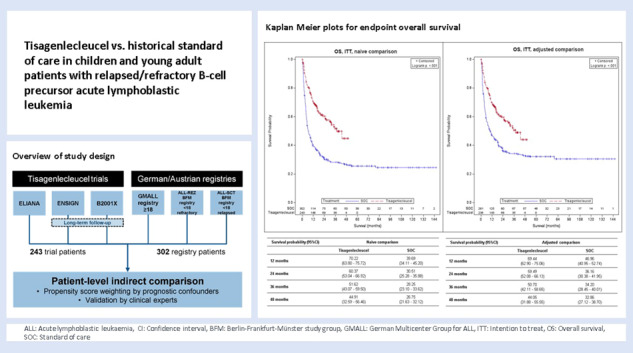

## Introduction

Acute lymphoblastic leukemia (ALL) is the most common malignancy in childhood [1, 2]. Overall survival (OS) exceeds 90% with contemporary multimodal chemotherapy, and allogeneic stem cell transplantation (HSCT) in selected patients with high-risk features [1–5]. About 15% of patients suffer a relapse of the disease requiring intensified salvage chemotherapy and allogeneic HSCT in most cases [6–8]. Patients with refractory diseases or second/more advanced relapse have a dismal prognosis and require new and innovative treatment approaches.

In 2018, the chimeric antigen receptor T-cell therapy tisagenlecleucel (Kymriah®) was authorized by the European Medicines Agency for the treatment of pediatric and young adult patients up to and including 25 years of age with B-cell ALL that is refractory, in relapse post-transplant, or in second or later relapse [9], thus focusing on the most unfavorable types of relapses. The efficacy and safety of tisagenlecleucel in this population was evaluated in the pivotal study ELIANA (CCTL019B2202; NCT02435849) and further supportive open-label, single-arm studies ENSIGN (CCTL019B2205J; NCT02228096) and CCTL019B2001X (NCT03123939), showing promising results for the CD19+ chimeric antigen receptor T-cell therapy in r/r ALL.

However, no comparative evidence from randomized controlled trials (RCT) is available due to the rarity and severity of the disease, the absence of a satisfactory standard regimen and the resulting ethical challenges for a RCT. To compare the efficacy of tisagenlecleucel with historical SOC, data from registries was analyzed in German speaking countries (Germany, Austria). The geographic focus and statistical methods were designed for use in health technology assessment by German authorities [10].

Especially in the era of personalized medicine and the development of novel drugs for rare diseases, single-arm studies are more common. Undertaking RCTs in orphan disease settings is often challenging and therefore calls for alternative methods of evidence generation. Indirect comparisons are increasingly becoming an integral part in health technology assessments as well as regulatory approval [11–13]. Specifically, individual patient data (IPD) from pivotal trials is often compared with published aggregated data as historical controls, known as matching-adjusted indirect comparison. In the treatment of r/r ALL, several indirect comparisons have been published [14–16] using aggregated data as controls. Using IPD in both arms allows for superior control of confounders via best practice analytical, decision and adjustment steps [17] but is often impossible due to non-availability of patient-level data in practice.

This study provides a non-randomized patient-level comparison of the efficacy of tisagenlecleucel and standard of care (SOC) among pediatric and young adult patients with r/r ALL. Unlike existing publications on indirect comparisons in r/r ALL evaluating the effects of tisagenlecleucel treatment, this study is the first efficacy comparison of tisagenlecleucel with real-world historical SOC using patient-level data for both intervention and comparator and thus following best practices for adjusted indirect comparisons [17].

## Materials and methods

### Study design and data sources

This is a retrospective, non-randomized study comparing tisagenlecleucel with historical SOC in pediatric and young adult patients with r/r B-cell precursor ALL, based on IPD for both intervention and historical control patients. For tisagenlecleucel, the comparison was based on pooled patient-level data from the pivotal study ELIANA (NCT02435849) as well as ENSIGN (NCT02228096) and CCTL019B2001X (NCT03123939) studies, including data from Long-Term Follow Up (LTFU) (NCT02445222) for ENSIGN and CCTL019B2001X (hereafter: tisagenlecleucel). As historical control arm (hereafter: SOC) pooled patient-level data from three registries in Germany and Austria (ALL-REZ BFM registry Berlin, GMALL registry Frankfurt am Main, ALL-SCT BFM registry Vienna) were used. Table 1 provides a detailed overview of the identified studies and registries used for this analysis.

**Table 1:** Overview of tisagenlecleucel studies and healthcare data provided by registries/study groups.

Data sources	Patient population	*N*
Studies*		
ELIANA (NCT02435849)	B-cell ALL between the age of 3 at screening to 21 years at the time of initial diagnosis (primary refractory or chemorefractory, second or later BM relapse or any BM relapse after allogeneic HSCT or otherwise ineligible for an allogeneic HSCT)	97
ENSIGN (NCT02228096)^a^	B-cell ALL and lymphoblastic lymphoma between the age of 3 at screening to 21 years at the time of initial diagnosis (primary refractory or chemorefractory, second or later BM relapse or any BM relapse after allogeneic HSCT or otherwise ineligible for an allogeneic HSCT)	75
CCTL019B2001X (NCT03123939)^a^	B-cell ALL up to and including 25 years of age at the time of screening (primary refractory or chemorefractory, second or later BM relapse or any BM relapse after allogeneic HSCT or otherwise ineligible for an allogeneic HSCT	73^b^
Registries/study groups*		
ALL-REZ BFM registry (Berlin)	Children and adolescents <18 years refractory to therapy (e.g., chemotherapy) or with a relapse after two lines of therapy (e.g. chemotherapy)	115
ALL-SCT BFM registry (Vienna)	Children and adolescents <18 years with a relapse after HSCT	104
GMALL registry (Frankfurt)	Adults ≥18 years refractory to standard induction chemotherapy or with relapse after HSCT, or refractory relapse or second relapse without prior SCT	83

*ALL* Acute lymphoblastic leukemia, *BFM* Berlin-Frankfurt-Münster, *BM* Bone marrow, *EMA* European Medicines Agency, *GMALL* German Multicenter Study Group for Adult ALL, *HSCT* Hematopoietic stem cell transplantation, *LTFU* Long-term follow up.

^a^including data from LTFU for ENSIGN (*N* = 31) and CCTL019B2001X (*N* = 42).

^b^excluding patients not fulfilling inclusion criteria, i.e., age <3 years.

*Time periods:

ELIANA: Data cut from July 01, 2019.

ENSIGN; LTFU: Final data cut from May 24, 2019; Data cut from May 2020 for EMA submission.

CCTL019B2001X; LTFU: Data until last patient last visit on 13 October 2020; Data cut from May 2020 for EMA submission.

ALL-REZ BFM: Included until September 2017 with longest possible follow-up period, but at least until end of 2019.

ALL-SCT BFM: Recruitment (last patient in) until 2013 with longest possible follow-up period, Data cut from 2017.

GMALL: Included until September 2017 with longest possible follow-up period, but at least until end of 2019.

### Study populations

The examined patient population consisted of pediatric and young adult patients from 3 up to and including 25 years with B-cell precursor r/r ALL. To ensure comparability between tisagenlecleucel and SOC populations, selection of patients from the SOC population were aligned to the inclusion and exclusion criteria of the pivotal study ELIANA as far as data availability allowed. These criteria were discussed with clinical experts and adapted to real-world requirements.

Treatment effects were assessed in two analysis sets: FAS (full analysis set) and ITT (intention to treat). FAS refers to tisagenlecleucel-infused patients, ITT refers to enrolled patients in tisagenlecleucel studies including those who subsequently received tisagenlecleucel infusion and those who did not. Both FAS and ITT populations were compared to SOC.

### Efficacy endpoints

Tisagenlecleucel and SOC were compared in terms of efficacy as represented by the endpoints OS, event-free survival (EFS), relapse-free survival (RFS) and overall remission rate (ORR). Due to the retrospective nature of this comparison, the definition of endpoints as primary or secondary was omitted. As far as available, patient characteristics and endpoint definitions for historical SOC were aligned to those used in tisagenlecleucel studies.

For time-to-event endpoints, hazard ratios from univariate cox-regression model along with 95% confidence intervals (CI), median follow-up with 95% CI (reverse KM method), survival probabilities with 95% CI and Kaplan–Meier curves with log-rank tests were estimated. For ORR, odds ratios (OR) were estimated by logistic regression, along with 95% CI and Wald Z-test.

For OS and ORR, complete data was available from all registries. For EFS, limited data was available from ALL-REZ BFM and ALL-SCT BFM registries and no data was available from GMALL registry (adult patients). For RFS, no data was available from ALL-SCT BFM and GMALL registries. These patients without response data were censored at day 1 in EFS analysis and not included in RFS analysis.

Complete survival data was available for both ITT and FAS tisagenlecleucel populations, allowing for a comparison of both. Response and relapse data was only available for infused tisagenlecleucel patients, restricting meaningful comparisons of ORR, EFS, and RFS to the FAS population. ORR was analyzed for both ITT and FAS populations by conservatively considering all non-infused patients as non-responders.

### Statistical approach

As this comparison was designed to be used in health technology assessment by German responsible authorities, all analyses were pre-specified based on the applicable methodological framework [10] and were outlined in a statistical analysis plan.

#### Line selection

For patients in the SOC population, multiple lines of therapy could meet the inclusion and exclusion criteria while patients in the tisagenlecleucel arm of this comparison received the study intervention in a specific line of therapy. A line selection procedure for SOC patients was thus pre-specified and performed to ensure inclusion of all SOC patients, each with exactly one line of therapy, while approximating the marginal distribution of treatment lines among tisagenlecleucel patients as best possible. The same procedure was used by Maziarz et al. [18].

#### Naïve and adjusted comparison

In non-randomized settings, the evaluation of a treatment on the outcome can be impacted by confounding bias. In order to avoid this bias, appropriate adjustment procedures were used to approximate the structural equality of the treatment groups. Therefore, potential confounders were identified via systematic literature review and selected according to prognostic relevance based on a structured discussion with clinical experts from the three participating registries prior implementing adjustment (Table 1). Those confounders were included for analyses if at least 80% of the patients in each treatment arm showed valid values in at least one therapy line for the confounder. Relevant confounders, which were controlled for, are displayed in the supplement in Tables 1 & 2.

For OS and ORR, analyses were performed as both naïve and adjusted comparisons by means of IPD meta-analysis considering the data source as random effect. As no randomization was applied, sufficient structural equality between the populations was achieved by a propensity score approach using fine stratification weights to minimize the potential effects of confounding and to obtain an unbiased estimate of treatment effect on the endpoints according to methods of the Institute for Quality and Efficiency in Healthcare (IQWiG) [10] and Desai & Franklin [17]. Density plots of the distribution of unweighted and weighted propensity score were generated to assess the distributional overlap between both treatment groups (see supplement Fig. 1 & 2). The overlapping patient populations are considered for the adjusted comparison, while non-overlapping ones are trimmed. This explains the difference in the number of patient populations between the naïve and adjusted comparison. An assessment of the balance of prognostic factors between the selected populations was conducted by comparing standardized mean differences (SMD) computed for each covariate in the weighted logistic regression (see supplement Table 1 & 2 and Fig. 3 & 4). A range of −0.25 to 0.25 was considered acceptable to indicate sufficient balance of confounders. This was a pre-specified acceptability threshold based on literature [19]. Otherwise, ISMDI > 0.25 would indicate serious imbalance. Should this especially apply to the SMDs after adjustment, sufficient overlap of patients would not be provided.

For EFS and RFS, confounders could not be adequately balanced using the pre-specified adjustment approach due to limited data availability from the registries. Results of the naïve comparison are thus presented.

For all calculated differences between treatment groups, two sided 95% CIs were reported. Statistical significance was calculated on a level of 0.05 using an appropriate two-sided statistical test without adjustment for multiplicity. The statistical analysis was performed using the SAS version 9.4 (SAS Institute Inc., Cary, NC, USA) and SAS-macros provided by Desai et al. [17].

#### Subgroup analysis

Subgroup analyses were performed for the endpoints OS and ORR. In this publication, results are shown for the endpoint OS (FAS/ITT) via forest plot. Analyses were done for subgroups that were predefined in the ELIANA study and were available in the SOC population.

## Results

### Baseline characteristics

Naïve and adjusted baseline characteristics for FAS tisagenlecleucel and SOC population are presented in Table 2 (ITT in supplement Table 3). In the naïve setting, the study comprised 243 ITT patients from the tisagenlecleucel study population, 209 of which received a tisagenlecleucel infusion (86%, FAS population), and 302 patients in SOC. Prior to adjustment, confounders were already mostly well or reasonably balanced in terms of SMDs, generally indicating similarity in demographics between treatment groups. Only the following confounders showed unacceptable balance prior to adjustment: age at first diagnosis (SMD = −0.4), status of disease-refractory to previous line of therapy (SMD = −0.3), status of disease-relapsed after previous line of therapy (SMD = 0.3) and baseline extramedullary disease presence (SMD = 0.3) (see supplement Table 1 and Fig. 3). In the adjusted FAS analysis set, 201 patients in the tisagenlecleucel and 273 in the SOC population were included. After the adjustment, all baseline confounders were well very balanced between the tisagenlecleucel and the SOC population, i.e., all included baseline confounders showed absolute ISMDI ≤ 0.1, which is significantly lower than the pre-specified acceptability threshold defined based on Stuart et al. [19] and indicates very good comparability of patient populations (see supplement Table 1 and Fig. 3).

**Table 2:** Baseline parameters for tisagenlecleucel and SOC, naïve and adjusted comparison, FAS.

Parameter		Tisagenlecleucel	SOC	Tisagenlecleucel	SOC
		(*N* = 209)	(*N* = 302)	(*N* = 201)	(*N* = 273)
		Naive comparison		Adjusted comparison	
Region- *n* (%)					
	EU	74 (35.4%)	301 (99.7%)	68 (33.8%)	272 (99.7%)
	US	125 (59.8%)	1 (0.3%)	123 (61.2%)	1 (0.3%)
	ROW	10 (4.8%)	0 (0.0%)	10 (5.0%)	0 (0.0%)
Gender- *n* (%)					
	female	94 (45.0%)	108 (35.8%)	92 (45.8%)	86 (31.7%)
	male	115 (55.0%)	194 (64.2%)	109 (54.2%)	187 (68.3%)
Age at first diagnosis- (years)					
	*N*	209	302	201	273
	Missing values	0	0	0	0
	Mean	8	10	8	8
	Standard deviation	5	7	5	6
	Median	6	9	6	6
	Minimum	0	0	0	0
	Maximum	21	25	21	25
Age at first diagnosis (</>10 years)- *n* (%)					
	<10 years	133 (63.6%)	167 (55.3%)	129 (64.2%)	190 (69.7%)
	≥10 years	76 (36.4%)	135 (44.7%)	72 (35.8%)	83 (30.3%)
Status of disease- *n* (%)					
	refractory to previous line of therapy	41 (19.6%)	96 (31.8%)	40 (19.9%)	57 (20.8%)
	relapsed after previous line of therapy	168 (80.4%)	206 (68.2%)	161 (80.1%)	216 (79.2%)
Time from initial diagnosis to first relapse- *n* (%)					
	<18 months	46 (22.0%)	64 (21.2%)	44 (21.9%)	46 (17.0%)
	18–36 months	68 (32.5%)	66 (21.9%)	66 (32.8%)	72 (26.5%)
	>36 months	80 (38.3%)	95 (31.5%)	78 (38.8%)	96 (35.3%)
	n.a.	15 (7.2%)	77 (25.5%)	13 (6.5%)	58 (21.2%)
Time from previous CR to relapse- (days)					
	*N*	0	176	0	175
	Missing values	209	126	201	98
	Mean	.	546	.	540
	Standard deviation	.	385	.	403
	Median	.	427	.	394
	Minimum	.	36	.	36
	Maximum	.	1806	.	1806
Previous HSCT- *n* (%)					
	No	93 (44.5%)	123 (40.7%)	87 (43.3%)	123 (45.1%)
	Yes	116 (55.5%)	179 (59.3%)	114 (56.7%)	150 (54.9%)
Number of previous lines of therapies- *n* (%)					
	1	19 (9.1%)	24 (7.9%)	15 (7.5%)	22 (7.9%)
	2	76 (36.4%)	127 (42.1%)	74 (36.8%)	105 (38.3%)
	>2	114 (54.5%)	151 (50.0%)	112 (55.7%)	147 (53.8%)
Number of previous relapses- *n* (%)					
	0	0 (0.0%)	24 (7.9%)	0 (0.0%)	22 (7.9%)
	1	0 (0.0%)	99 (32.8%)	0 (0.0%)	51 (18.7%)
	2	0 (0.0%)	131 (43.4%)	0 (0.0%)	142 (52.1%)
	≥3	0 (0.0%)	48 (15.9%)	0 (0.0%)	58 (21.3%)
	n.a.	209 (100.0%)	0 (0.0%)	201 (100.0%)	0 (0.0%)
Morphologic blast count in BM- *n* (%)					
	Low (<50%)	69 (33.0%)	74 (24.5%)	67 (33.3%)	70 (25.8%)
	High (≥50%)	137 (65.6%)	72 (23.8%)	131 (65.2%)	77 (28.2%)
	n.a.	3 (1.4%)	156 (51.7%)	3 (1.5%)	126 (46.0%)
Hypodiploidy- *n* (%)					
	No or n.a.	205 (98.1%)	301 (99.7%)	200 (99.5%)	273 (100.0%)
	Yes	4 (1.9%)	1 (0.3%)	1 (0.5%)	0 (0.0%)
BCR-ABL- *n* (%)					
	negative or n.a.	200 (95.7%)	289 (95.7%)	193 (96.0%)	265 (97.1%)
	positive	9 (4.3%)	13 (4.3%)	8 (4.0%)	8 (2.9%)
MLL rearrangement- *n* (%)					
	No or n.a.	203 (97.1%)	293 (97.0%)	196 (97.5%)	264 (96.7%)
	Yes	6 (2.9%)	9 (3.0%)	5 (2.5%)	9 (3.3%)
Baseline extramedullary disease presence- *n* (%)					
	No or n.a.	184 (88.0%)	292 (96.7%)	180 (89.6%)	240 (88.1%)
	Yes	25 (12.0%)	10 (3.3%)	21 (10.4%)	33 (11.9%)
Karnofsky-Index- *n* (%)					
	20	0 (0.0%)	1 (0.3%)	0 (0.0%)	1 (0.2%)
	40	0 (0.0%)	3 (1.0%)	0 (0.0%)	3 (1.1%)
	50	5 (2.4%)	5 (1.7%)	5 (2.5%)	3 (1.2%)
	60	9 (4.3%)	7 (2.3%)	7 (3.5%)	10 (3.5%)
	70	16 (7.7%)	11 (3.6%)	15 (7.5%)	13 (4.7%)
	80	36 (17.2%)	22 (7.3%)	35 (17.4%)	24 (8.7%)
	90	70 (33.5%)	16 (5.3%)	70 (34.8%)	18 (6.6%)
	100	73 (34.9%)	21 (7.0%)	69 (34.3%)	24 (8.7%)
	n.a.	0 (0.0%)	216 (71.5%)	0 (0.0%)	179 (65.4%)

*BM* Bone marrow, *CR* Complete remission, *EU* European Union, *FAS* Full analysis set, *HSCT* Hematopoietic stem cell transplantation, *MLL* Mixed-lineage leukemia, n.a: not available, *ROW* Rest-of-the-world, *SOC* Standard of care, *US* United States.

The adjusted values refer to the weighted populations using fine stratification weights after trimming of patients in non-overlapping regions of the propensity score distribution.

Unless otherwise specified, parameters are evaluated for tisagenlecleucel data (ELIANA, ENSIGN incl. LTFU, CCTL019B2001X incl. LTFU) at the screening time and at the time of the qualifying event for the historical control. The qualifying events were defined as follows: primary refractory or chemorefractory after relapse, second or greater BM or any BM relapse after HSCT.

### Efficacy endpoints

Treatment with tisagenlecleucel was associated with a significantly lower hazard of death in both ITT and FAS analyses [Hazard ratio (HR)_ITT_ = 0.54 (0.41–0.71), *p* < 0.001; HR_FAS_ = 0.47 (0.35–0.62), *p* < 0.001] vs. SOC in the adjusted comparison. The adjusted OS_ITT_ survival probability at 2 years was 59.49% (52.08–66.13%) for tisagenlecleucel vs. 36.16% (30.38–41.95%) for SOC population and 65.41% (58.02–71.82%) vs. 36.83% (30.98–42.68%) in adjusted OS_FAS_. The median follow-up was 22.5 months (18.0–29.1) vs. 60.5 months (41.1–70.9) and 30.2 months (28.1–34.9) vs. 60.5 months (48.2–75.3) for OS_ITT_ and OS_FAS_, respectively.

Tisagenlecleucel therapy was associated with a significantly higher overall response, with an adjusted OR_ITT_ of 1.99 (1.33–2.97, *p* < 0.001) and OR_FAS_ of 3.34 (2.14–5.19, *p* < 0.001) compared with the SOC population.

The naïve median follow-up in EFS_FAS_ was 21.2 months (12.4–23.7) for tisagenlecleucel and 69.9 months (48.9–92.9) for SOC. Hazard of event was 33% significantly lower in the tisagenlecleucel population than in the SOC population [HR_FAS_ = 0.67 (0.52–0.86)]. The naïve EFS_FAS_ survival probability at 2 years was 42.31% (34.55–49.85%) vs. 30.23% (24.01–36.68%).

For naïve RFS, therapy in tisagenlecleucel population was non-significantly associated with an HR_FAS_ of 0.77 (0.51–1.18). The RFS_FAS_ survival probability at 2 years was 59.60% (49.74–68.16%) vs. 54.57% (42.60–65.05%), respectively. The median follow-up was 13.7 (11.3–21.4) months for tisagenlecleucel and 73.7 (59.1–102.2) months for SOC.

**Fig. 1:**
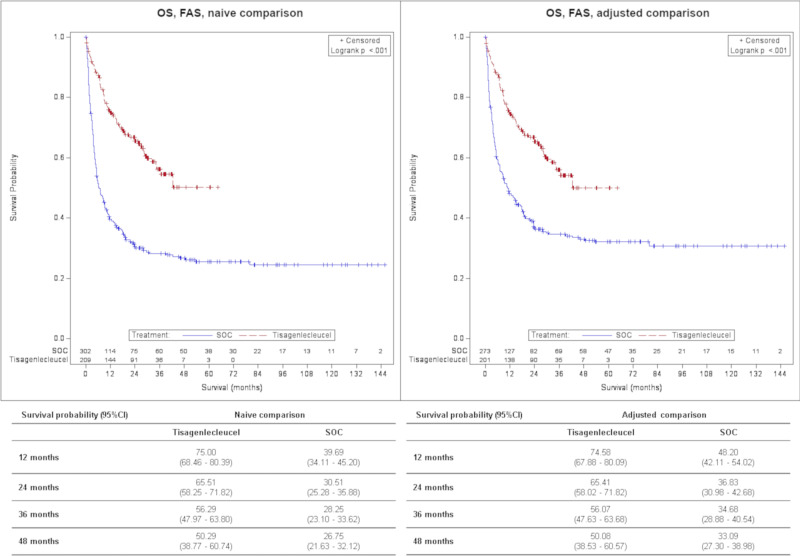
Comparison of OS for tisagenlecleucel vs. SOC, FAS, Kaplan–Meier plot. CI Confidence interval, FAS Full analysis set, OS Overall survival, SOC Standard of care.

**Fig. 2:**
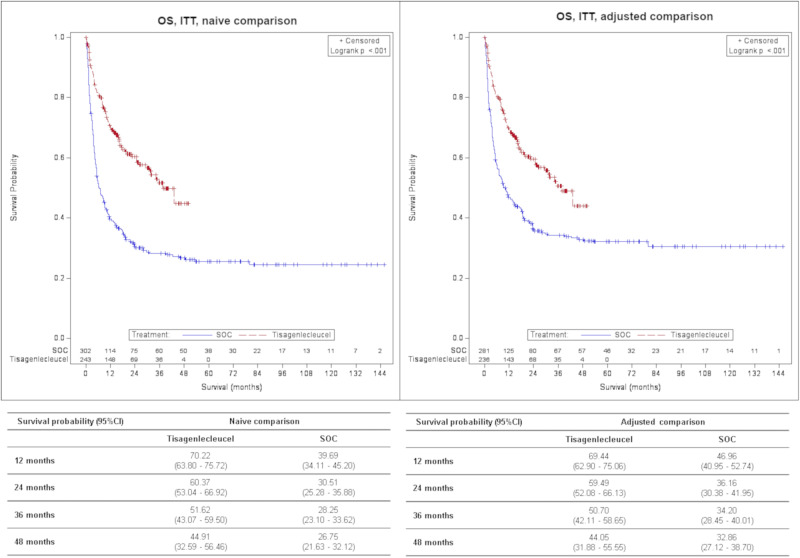
Comparison of OS for tisagenlecleucel vs. SOC, ITT, Kaplan–Meier plot. CI Confidence interval, ITT Intention to treat, OS Overall survival, SOC Standard of care.

**Fig. 3:**
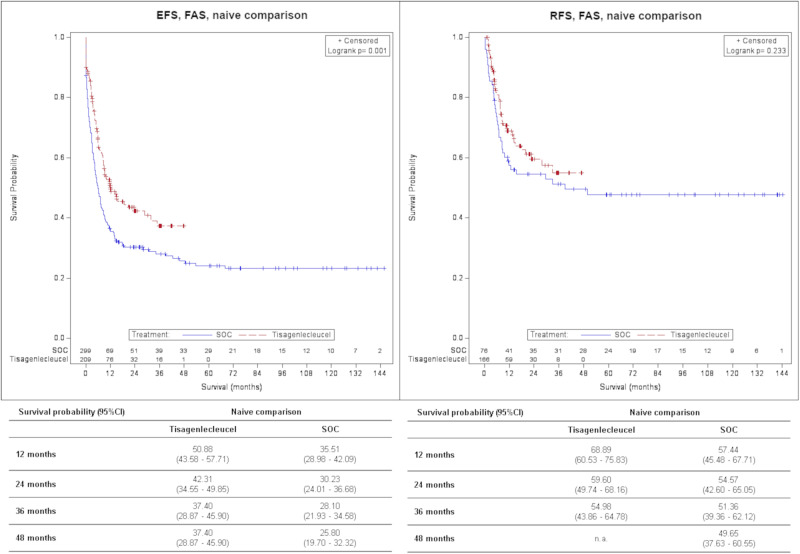
Comparison of EFS and RFS for tisagenlecleucel vs. SOC, FAS, Kaplan–Meier plot. CI Confidence interval, EFS Event-free survival, FAS Full analysis set, RFS Relapse-free survival, SOC Standard of care.

An overview of efficacy results is displayed in Table 3. Survival probabilities for OS, EFS and RFS at 1, 2, 3 and 4 years are shown in Figs. 1–3. The median follow-up for OS, EFS and RFS is presented in the supplement Table 4.

**Table 3:** Overview of results – Tisagenlecleucel vs. SOC.

Tisagenlecleucel vs. SOC		
FAS	Naïve comparison	Adjusted comparison
	N(Tisagenlecleucel) = 209	N(Tisagenlecleucel) = 201
	N(SOC) = 302	N(SOC) = 273
*OS*	0.38 (0.29–0.49); <0.001	0.47 (0.35–0.62); <0.001
Univariate Cox-Regression		
HR (95 % CI); *p*-value		
Defined as the time from the date of treatment start to the date of death due to any cause.		
ORR*	4.95 (3.30–7.43); <0.001	3.34 (2.14–5.19); <0.001
Logistic Regression		
OR (95% CI)		
Defined as the proportion of patients with a best overall disease response of CR or CRi.		
*EFS*^a^*	0.67 (0.52–0.86); 0.001	n.a.
Univariate Cox-Regression		
HR (95 % CI); *p*-value		
Defined as the time from the date of treatment start to the date of relapse, death due to any cause after remission or treatment failure, whatever occurs first.		
*RFS*^*b*^	0.77 (0.51–1.18); 0.233	n.a.
Univariate Cox-Regression		
HR (95 % CI); *p*-value		
Defined as time from achievement of CR or CRi whatever occurs first to date of relapse or death due to any cause during CR or CRi.		
*ITT*	Naïve comparison	*Adjusted comparison*
	N(Tisagenlecleucel) = 243	N(Tisagenlecleucel) = 236
	N(SOC) = 302	N(SOC) = 281
*OS*	0.44 (0.34–0.56); <0.001	0.54 (0.41–0.71); <0.001
Univariate Cox-Regression		
HR (95% CI); *p*-value		
SOC: Defined as the time from the date of treatment start to the date of death due to any cause		
Tisagenlecleucel: Defined as the time from the date of enrollment to the date of death due to any cause.		
ORR*	2.76 (1.94–3.94); <0.001	1.99 (1.33–2.97); <0.001
Logistic Regression		
OR (95% CI)		
Defined as the proportion of patients with a best overall disease response of CR or CRi.		

*CI* Confidence interval, *CR* Complete remission, *CRi* CR with incomplete blood count recovery, *EFS* Event-free survival, *FAS* Full analysis set, *HR* Hazard ratio, *ITT* Intention to treat, *n.a*. not available, *OR* Odds ratio, *ORR* Overall remission rate, *OS* Overall survival, *RFS* Relapse-free survival, *SOC* Standard of care.

^a^Data not available in the GMALL dataset (Frankfurt). Patients from this registry were included but censored at day 1.

^b^Only data from the ALL-REZ BFM registry (Berlin) was included.

*missings SOC, naïve *N* = 3.

*p*-value: based on Log-Rank Test; *p*-value (ORR): based on Z-test.

### Subgroup analysis

**Fig. 4:**
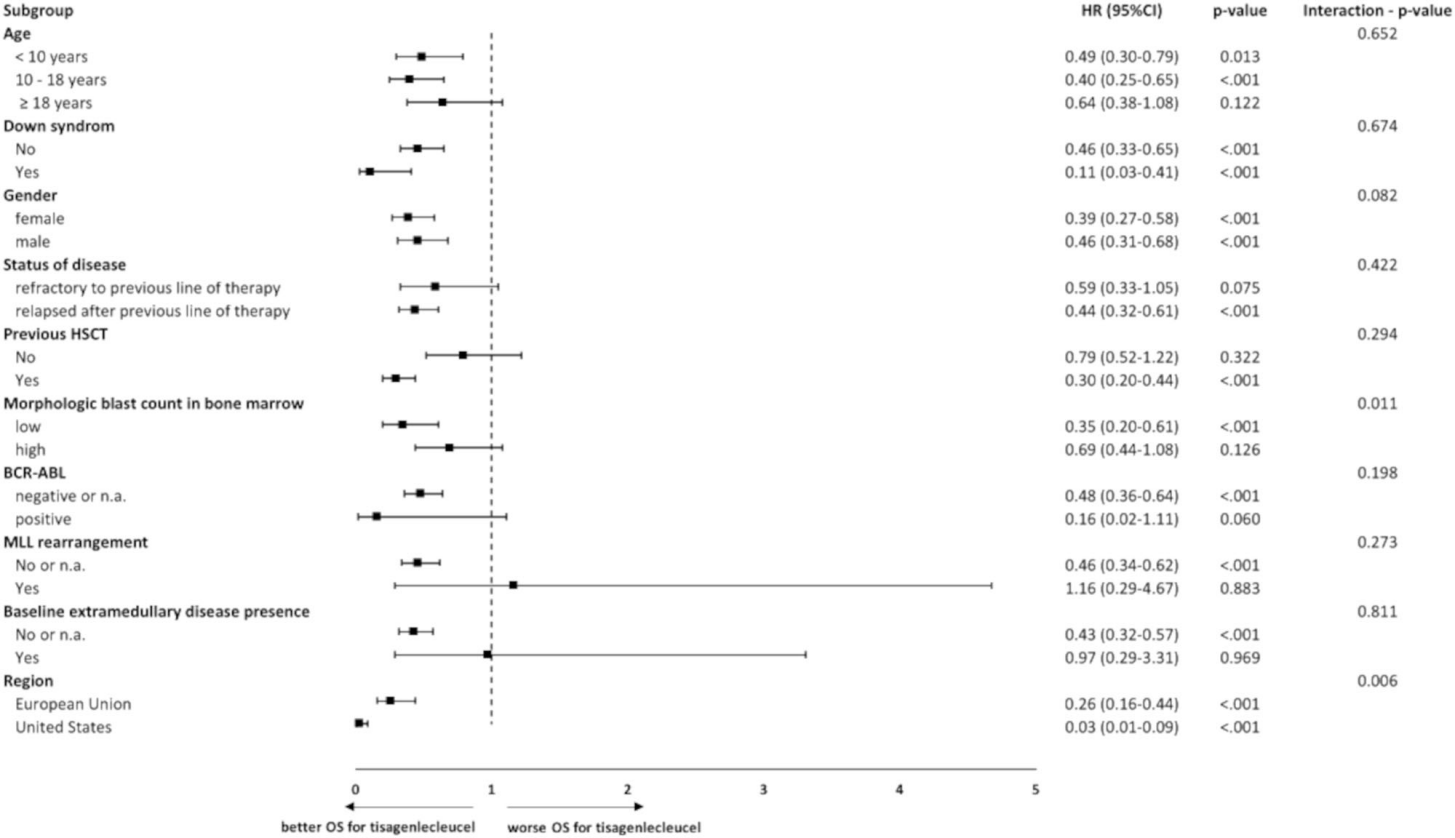
Comparison of OS for tisagenlecleucel vs. SOC according to subgroup categories, adjusted comparison, Forest plot, FAS. CI Confidence interval, HR Hazard ratio, HSCT Hematopoietic stem cell transplantation, MLL Mixed-lineage leukemia, n.a. not available, OS Overall survival. Note: For this subgroup analysis fine stratification weights were applied from main analysis; only subgroups for which patients were available in both arms are presented; *p*-value calculated by log-rank test; interaction *p*-value calculated by using likelihood ratio test.

**Fig. 5:**
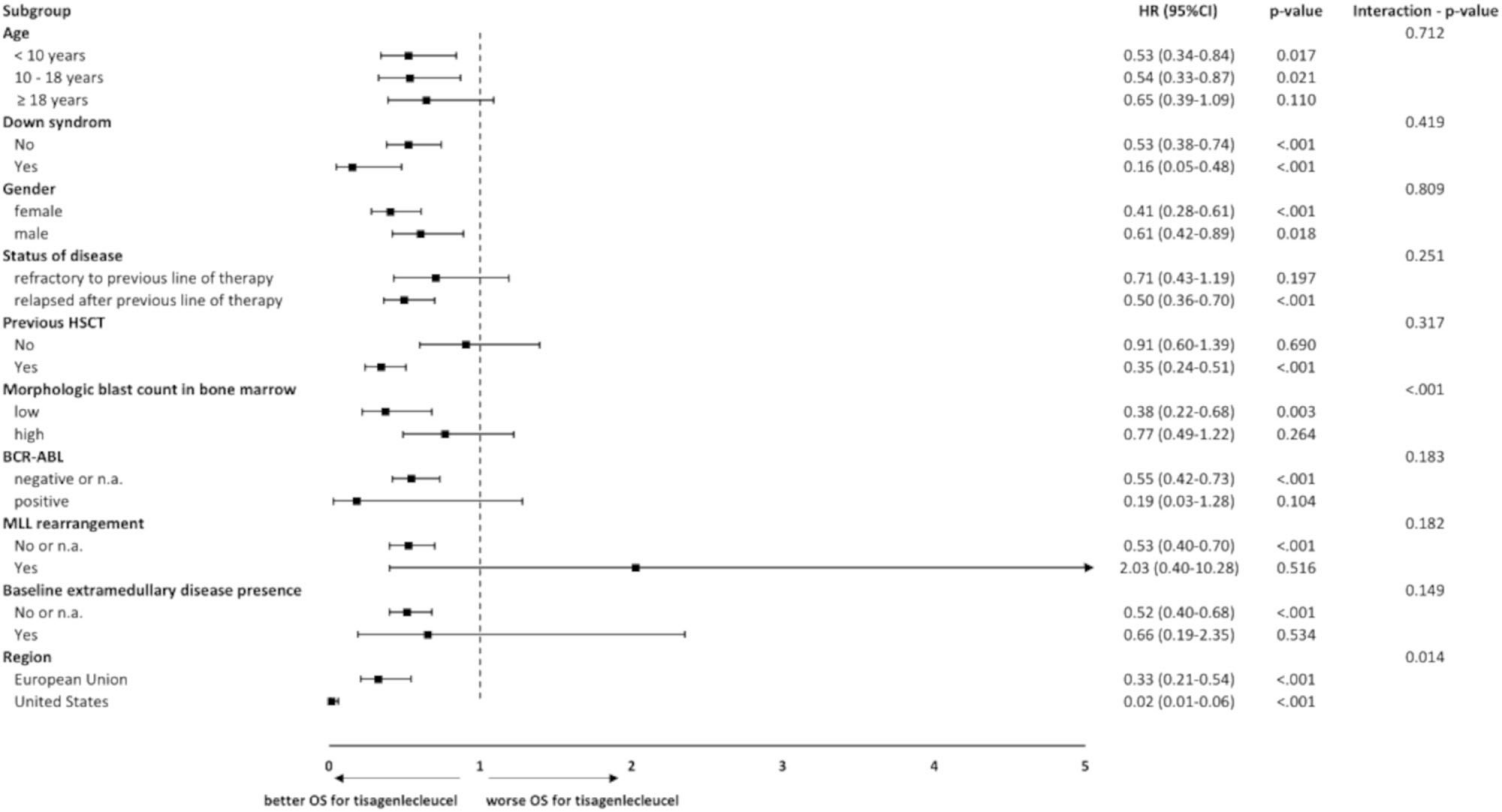
Comparison of OS for tisagenlecleucel vs. SOC according to subgroup categories, adjusted comparison, Forest plot, ITT. CI Confidence interval, HR Hazard ratio, HSCT Hematopoietic stem cell transplantation, MLL Mixed-lineage leukemia, n.a. not available, OS Overall survival. Note: For this subgroup analysis fine stratification weights were applied from main analysis; only subgroups for which patients were available in both arms are presented; *p*-value calculated by log-rank test; interaction *p*-value calculated by using likelihood ratio test.

In Figs. 4 and 5 adjusted results are presented in a forest plot for OS. The findings from the subgroup analysis were consistent with the results from the main analysis. All subgroup HR except mixed-lineage leukemia (MLL) rearrangement (yes) favor tisagenlecleucel treatment.

## Discussion

The present study utilized pooled IPD from three single-arm trials for tisagenlecleucel as well as IPD from three registries from Germany and Austria to compare tisagenlecleucel vs. SOC in terms of efficacy as represented by the endpoints OS, EFS, RFS and ORR. To the authors’ knowledge, this is the first efficacy comparison of tisagenlecleucel with real-world historical SOC using patient-level data for both intervention and comparator and thus following best practices for adjusted indirect comparisons [17]. Therefore, this publication adds supporting evidence that tisagenlecleucel is superior to SOC based on the best possible adjustment for confounders in indirect comparisons and very comparable cohorts. Homogeneity of treatment arms (in terms of ISMDI ≤ 0.25), indicates that already prior adjustment, 7 out of 11 pre-specified confounders were reasonably balanced indicating that defining inclusion and exclusion criteria for SOC patients based on those in the ELIANA trial lead to comparable patients being included in the study. After adjustment, very good balance of all relevant pre-specified confounders was achieved, and all 11 pre-specified confounders show SMDs <0.1. Results from adjusted analyses are thus based on highly comparable cohorts.

Results showed favorable outcomes for tisagenlecleucel compared to SOC for all examined endpoints. HR for OS_ITT,adjusted_, EFS_FAS,naïve_ and RFS_FAS,naïve_ were 0.54 (0.41–0.71, *p* < 0.001), 0.67 (0.52–0.86, *p* = 0.001), and 0.77 (0.51–1.18, *p* = 0.233), respectively. In RFS only data from ALL-REZ BFM registry was included due to lacking data in the other two registries which may explain the non-significance. The OS_ITT,adjusted_, EFS_FAS,naïve_ and RFS_FAS,naive_ survival probability at 2 years was 59.49% for tisagenlecleucel vs. 36.16% for SOC population, 42.31% vs. 30.23% and 59.60% vs. 54.57%, respectively. OR for ORR_ITT,adjusted_ was 1.99 (1.33–2.97, *p* < 0.001). The median follow-up time was 22.5 months for tisagenlecleucel vs. 60.5 months for SOC population for OS_ITT,adjusted_, 21.2 months vs. 69.9 months for EFS_FAS,naïve_ and 13.7 months vs. 73.7 months for RFS_FAS,naïve_.

Although lacking comparative evidence from RCTs, tisagenlecleucel has been approved by EMA based on a phase II single-arm trial. With regard to the rising approval of cell and gene therapies, there will be more studies of such kind, evaluating orphan indications where RCTs cannot be applied and new evidence standards need to be incorporated. Not only IQWiG, but also EMA emphasizes the need for indirect comparisons, even though rarity of diseases will present challenges in the comparability of patient populations [13, 20, 21].

Generally, published indirect comparisons of tisagenlecleucel for ALL are limited so far. The study of Ma et al. examined clinical benefits of tisagenlecleucel compared with aggregated data from historical SOC regimens in r/r pediatric and young adult ALL patients in the form of a matching-adjusted indirect comparison. Thereby, baseline covariates are adjusted for differences by using IPD from trials of one treatment and aggregated data from other trials (usually the control arm). This study presented promising outcomes for tisagenlecleucel in terms of prolonged OS compared with the included historical SOC regimens (blinatumomab, clofarabine monotherapy, clofarabine combination regimens and two salvage therapies). Hazard of death ranged from a 85% reduction [HR = 0.15 (0.09–0.25)] for salvage-1 and 68% reduction [HR = 0.32 (0.16–0.64)] for blinatumomab. OR ranged from 4.11 (1.84–9.21) for clofarabine plus etoposide plus cyclophosphamide combination therapy to 12.88 (5.02–33.04) for clofarabine monotherapy [14]. Results of this study were also in line with the favorable outcomes for tisagenlecleucel treatment found in our study. However, results from Ma et al. are not fully comparable due to aggregated data in the comparator arm resulting in limited adjustment for relevant confounders as well as cross trial differences. Moreover, the patient population in the control arm (patients until 17 years of age; not limited to B-cell ALL) differed from our study.

So far, Verneris et al. [22] was the only study incorporating IPD for the tisagenlecleucel as well as the comparator arm. The analysis estimated the treatment effect of tisagenlecleucel vs. blinatumomab comparing IPD from two pivotal trials, ELIANA and MT103-205, on rates of CR and OS in patients with r/r ALL. Treatment with tisagenlecleucel was associated with a statistically significant higher likelihood of achieving CR [OR = 3.83 (1.88–7.79)] and 60% lower hazard of death [HR = 0.40 (0.26–0.63)] when adjusting for prognostic factors [22]. These results supported our findings reporting a prolonged OS and a higher CR for tisagenlecleucel treatment, although the studies are not directly comparable as Verneris et al. only compared to blinatumomab +/- SCT. Further, we adjusted for additional confounders such as relapsed after previous line of therapy, previous lines of therapies, MLL rearrangement, hypodiploidy, BCR-ABL and baseline extramedullary disease presence. Furthermore, the analyses provided in our study went one step further in terms of alternative evidence generation to allow sufficient comparison: for the tisagenlecleucel arm, pooled evidence of ELIANA, ENSIGN and CCTL019B2001X (incl. LTFU) was provided. The inclusion of real-world data from registries for the control arm was able to further support the evidence of beneficial treatment with tisagenlecleucel. In order to minimize bias potential in our analyses, systematic identification of relevant confounders and overall pre-specification of planned analyses in a statistical analyses plan was performed.

The results of this study have to be interpreted in consideration with the following limitations. The results for the FAS population may overestimate the outcomes as only treated patients are included, not reflecting routine care in which patients may suffer from e.g. adverse events or early death and therefore fail to get treatment. Conversely, analyzing outcomes for all enrolled patients (ITT) rather than only including infused patients may lead to underestimation of results, for example due to shorter manufacturing times and improved manufacturing processes in routine care as compared to clinical studies. Thus, for OS and ORR both ITT and FAS results are shown. For ORR_ITT_ a conservative approach was chosen, namely including non-infused patients as non-responders.

For EFS and RFS, significant limitations to data availability existed for both tisagenlecleucel and SOC treatment arms. Response and relapse data was only available for infused tisagenlecleucel patients, limiting the comparison to the FAS population for tisagenlecleucel. Data for EFS was not available from GMALL registry (adults) and data on RFS was not available from both ALL-SCT BFM (after HSCT) and GMALL (adults) registries. Thus, confounders could not be adequately balanced between residual patient populations and only a naïve comparison could be performed. Results of this naïve comparison are reported but are potentially biased. Residual populations for EFS and RFS are almost exclusively pediatric patients, which are compared to the tisagenlecleucel population including young adults. RFS analyses by definition only included registry patients in CR/CRi, which are therefore generally eligible for SCT, whereas in the tisagenlecleucel population all infused patients were included. Any comparison between clinical data and real-world data may be biased by differences in visiting schedules potentially concerning EFS and RFS results in this analysis. Last, safety outcomes and health-related quality of life were not examined in this study as those are usually not sufficiently documented in a (German) registry setting.

The study investigated efficacy outcomes of tisagenlecleucel in a non-randomized comparison with IPD data from German/Austrian registries in pediatric patients and young adult patients with r/r ALL with a significant OS benefit. This is the best provided alternative method of evidence generation with regard to existing evidence of single-arm trials for tisagenlecleucel. The positive results generated in the study highlight the importance of tisagenlecleucel as a suitable therapeutic option for this patient population.

### Supplementary information

Supplemental material revised

## Data Availability

The data that support the findings of this study are available from Novartis Pharma GmbH but restrictions apply to the availability of these data, which were used under license for the current study, and so are not publicly available. Data are however available from the authors upon reasonable request and with permission of Novartis Pharma GmbH.
