# Supplementary material for: Tisagenlecleucel vs. historical standard of care in children and young adult patients with relapsed/refractory B-cell precursor acute lymphoblastic leukemia
Source: Leukemia. 2023 Oct 25;37(12):2346–55. doi: 10.1038/s41375-023-02042-4 (PMC10681894; doi:10.1038/s41375-023-02042-4)
Supplement: Supplementary file 1 — Supplemental material revised [file 41375_2023_2042_MOESM1_ESM.pdf]

Supplemental material

This file contains information on standardized mean differences as well as propensity score distribution before and after adjustment for relevant confounders (Full analysis set, FAS; Intention-to-treat, ITT). Baseline parameters are presented (ITT, naïve and adjusted). Median follow-up for OS, EFS and RFS are displayed along with 95% confidence intervals.

Tables

Supplementary Table 1: Standardized mean differences by confounder, FAS 3

Supplementary Table 2: Standardized mean differences by confounder, ITT 4

Supplementary Table 3: Baseline parameters for tisagenlecleucel and SOC, naïve and adjusted comparison, ITT 5

Supplementary Table 4: Median follow-up for OS, EFS and RFS 9

Figures

Supplementary Figure 1: Histogram and density plot, FAS, unweighted (left) and weighted (right) 10

Supplementary Figure 2: Histogram and density plot, ITT, unweighted (left) and weighted (right) 11

Supplementary Figure 3: Standardized mean differences, balance plot, unweighted and weighted, FAS 12

Supplementary Figure 4: Standardized mean differences, balance plot, unweighted and weighted, ITT 13

Supplementary Table 1: Standardized mean differences by confounder, FAS

Confounder	Standardized mean differences (unweighted)	Standardized mean differences (weighted)
Age at first diagnosis	-0.4	0
BCR-ABL	0	0.1
Hypodiploidy	0.2	0.1
MLL rearrangement	0	0
Status of disease - refractory to previous line of therapy	-0.3	0
Status of disease - relapsed after previous line of therapy	0.3	0
Baseline extramedullary disease presence	0.3	0
Previous HSCT	-0.1	0
Number of previous lines of therapies - 1	0	0
Number of previous lines of therapies - 2	-0.1	0
Number of previous lines of therapies - > 2	0.1	0

FAS: Full analysis set, HSCT: Hematopoietic stem cell transplantation, MLL: Mixed-lineage leukemia

Supplementary Table 2: Standardized mean differences by confounder, ITT

Confounder	Standardized mean differences (unweighted)	Standardized mean differences (weighted)
Age at first diagnosis	-0.3	-0.1
BCR-ABL	0	0
Hypodiploidy	0.2	0.2
MLL rearrangement	0	0
Status of disease - refractory to previous line of therapy	-0.3	0
Status of disease - relapsed after previous line of therapy	0.3	0
Baseline extramedullary disease presence	0.3	-0.1
Previous HSCT	-0.1	0
Number of previous lines of therapies - 1	0.1	0.1
Number of previous lines of therapies - 2	-0.1	0
Number of previous lines of therapies - > 2	0.1	0

HSCT: Hematopoietic stem cell transplantation, ITT: Intention to treat, MLL: Mixed lineage leukemia

Supplementary Table 3: Baseline parameters for tisagenlecleucel and SOC, naïve and adjusted comparison, ITT

**Parameter**		**Tisagenlecleucel (N=243)**	**SOC (N=302)**	**Tisagenlecleucel (N=236)**	**SOC (N=281)**
		**Naive comparison**		**Adjusted comparison**	
Region- n (%)					
	EU	83 (34.2%)	301 (99.7%)	77 (32.6%)	280 (99.7%)
	US	148 (60.9%)	1 (0.3%)	147 (62.3%)	1 (0.3%)
	ROW	12 (4.9%)	0 (0.0%)	12 (5.1%)	0 (0.0%)
Gender- n (%)					
	female	106 (43.6%)	108 (35.8%)	104 (44.1%)	94 (33.4%)
	male	137 (56.4%)	194 (64.2%)	132 (55.9%)	187 (66.6%)
Age at first diagnosis- (years)					
	N	243	302	236	281
	Missing values	0	0	0	0
	Mean	8	10	8	9
	Standard deviation	5	7	5	7
	Median	7	9	7	7
	Minimum	0	0	0	0
	Maximum	22	25	22	25
Age at first diagnosis (</>10 years)- n (%)					
	< 10 years	151 (62.1%)	167 (55.3%)	146 (61.9%)	191 (67.9%)
	≥ 10 years	92 (37.9%)	135 (44.7%)	90 (38.1%)	90 (32.1%)
Status of disease- n (%)					

Parameter		Tisagenlecleucel (N=243)	SOC (N=302)	Tisagenlecleucel (N=236)	SOC (N=281)
		Naive comparison		Adjusted comparison	
	refractory to previous line of therapy	49 (20.2%)	96 (31.8%)	49 (20.8%)	62 (21.9%)
	relapsed after previous line of therapy	194 (79.8%)	206 (68.2%)	187 (79.2%)	219 (78.1%)
Time from initial diagnosis to first relapse- n (%)					
	< 18 months	54 (22.2%)	64 (21.2%)	52 (22.0%)	51 (18.1%)
	18 - 36 months	84 (34.6%)	66 (21.9%)	82 (34.7%)	71 (25.4%)
	> 36 months	86 (35.4%)	95 (31.5%)	84 (35.6%)	97 (34.5%)
	n.a.	19 (7.8%)	77 (25.5%)	18 (7.6%)	62 (22.0%)
Time from previous CR to relapse (days)					
	N	0	176	0	175
	Missing values	243	126	236	106
	Mean	.	546	.	525
	Standard deviation	.	385	.	406
	Median	.	427	.	364
	Minimum	.	36	.	36
	Maximum	.	1806	.	1806
Previous HSCT- n (%)					
	No	110 (45.3%)	123 (40.7%)	105 (44.5%)	129 (45.7%)

Parameter		Tisagenlecleucel (N=243)	SOC (N=302)	Tisagenlecleucel (N=236)	SOC (N=281)
		Naive comparison		Adjusted comparison	
	Yes	133 (54.7%)	179 (59.3%)	131 (55.5%)	152 (54.3%)
Number of previous lines of therapies- n (%)					
	1	24 (9.9%)	24 (7.9%)	21 (8.9%)	21 (7.4%)
	2	86 (35.4%)	127 (42.1%)	85 (36.0%)	101 (36.0%)
	> 2	133 (54.7%)	151 (50.0%)	130 (55.1%)	159 (56.6%)
Number of previous relapses- n (%)					
	0	0 (0.0%)	24 (7.9%)	0 (0.0%)	21 (7.4%)
	1	0 (0.0%)	99 (32.8%)	0 (0.0%)	57 (20.4%)
	2	0 (0.0%)	131 (43.4%)	0 (0.0%)	135 (48.2%)
	≥3	0 (0.0%)	48 (15.9%)	0 (0.0%)	67 (24.0%)
	n.a.	243 (100.0%)	0 (0.0%)	236 (100.0%)	0 (0.0%)
Morphologic blast count in BM- n (%)					
	Low (<50%)	74 (30.5%)	74 (24.5%)	72 (30.5%)	71 (25.1%)
	High (≥50%)	166 (68.3%)	72 (23.8%)	161 (68.2%)	78 (27.8%)
	n.a.	3 (1.2%)	156 (51.7%)	3 (1.3%)	132 (47.1%)
Hypodiploidy- n (%)					
	No or n.a.	237 (97.5%)	301 (99.7%)	233 (98.7%)	281 (100.0%)
	Yes	6 (2.5%)	1 (0.3%)	3 (1.3%)	0 (0.0%)
BCR-ABL- n (%)					
	negative or n.a.	234 (96.3%)	289 (95.7%)	228 (96.6%)	274 (97.4%)
	positive	9 (3.7%)	13 (4.3%)	8 (3.4%)	7 (2.6%)

Parameter		Tisagenlecleucel (N=243)	SOC (N=302)	Tisagenlecleucel (N=236)	SOC (N=281)
		Naive comparison		Adjusted comparison	
MLL rearrange-ment- n (%)					
	No or n.a.	237 (97.5%)	293 (97.0%)	230 (97.5%)	271 (96.6%)
	Yes	6 (2.5%)	9 (3.0%)	6 (2.5%)	10 (3.4%)
Baseline extrame-dullary disease presence- n (%)					
	No or n.a.	215 (88.5%)	292 (96.7%)	212 (89.8%)	247 (87.9%)
	Yes	28 (11.5%)	10 (3.3%)	24 (10.2%)	34 (12.1%)
Karnofsky-Index-n (%)					
	20	0 (0.0%)	1 (0.3%)	0 (0.0%)	1 (0.4%)
	40	0 (0.0%)	3 (1.0%)	0 (0.0%)	3 (1.0%)
	50	5 (2.1%)	5 (1.7%)	5 (2.1%)	4 (1.5%)
	60	9 (3.7%)	7 (2.3%)	7 (3.0%)	11 (4.0%)
	70	16 (6.6%)	11 (3.6%)	16 (6.8%)	17 (6.1%)
	80	36 (14.8%)	22 (7.3%)	35 (14.8%)	25 (9.0%)
	90	70 (28.8%)	16 (5.3%)	70 (29.7%)	15 (5.3%)
	100	73 (30.0%)	21 (7.0%)	70 (29.7%)	20 (7.2%)
	n.a.	34 (14.0%)	216 (71.5%)	33 (14.0%)	184 (65.6%)

BM: Bone marrow, CR: Complete remission, EU: European union, HSCT: Hematopoietic stem cell transplantation, ITT: Intention to treat, MLL: Mixed-lineage leukemia, n.a: not available, ROW: Rest-of-the-world, SOC: Standard of care, US: United States

The adjusted values refer to the weighted populations using fine stratification weights after trimming of patients in non-overlapping regions of the propensity score distribution.

Unless otherwise specified, parameters are evaluated for tisagenlecleucel population (ELIANA, ENSIGN incl. LTFU, CCTL019B2001X incl. LTFU) at the screening time and at the time of the qualifying event for the external control. The qualifying events were defined as follows: primary refractory or chemorefractory after relapse, 2nd or greater BM or any BM relapse after allogeneic HSCT.

Supplementary Table 4: Median follow-up for OS, EFS and RFS

Median follow-up (in months) (95%CI)				
**FAS**				
	**Naïve comparison**		**Adjusted comparison**	
	**Tisagenlecleucel**	**SOC**	**Tisagenlecleucel**	**SOC**
**OS**	30.19 (27.30–33.08)	59.39 (48.24–70.88)	30.19 (28.06–34.91)	60.51 (48.24–75.31)
**EFS**	21.20 (12.40–23.66)	69.86 (48.93–92.90)	n.a.	n.a.
**RFS**	13.72 (11.26–21.39)	73.73 (59.13–102.15)	n.a.	n.a.
**ITT**				
	**Naïve comparison**		**Adjusted comparison**	
	**Tisagenlecleucel**	**SOC**	**Tisagenlecleucel**	**SOC**
**OS**	21.79 (17.00–26.64)	59.39 (48.24–70.88)	22.51 (18.01–29.11)	60.51 (41.05–70.88)

EFS: Event-free survival, FAS: Full analysis set, ITT: Intention-to-treat, n.a.: not available, OS: Overall survival, RFS: Relapse-free survival

Median follow-up was calculated according to reverse KM method.

Supplementary Figure 1: Histogram and density plot, FAS, unweighted (left) and weighted (right)

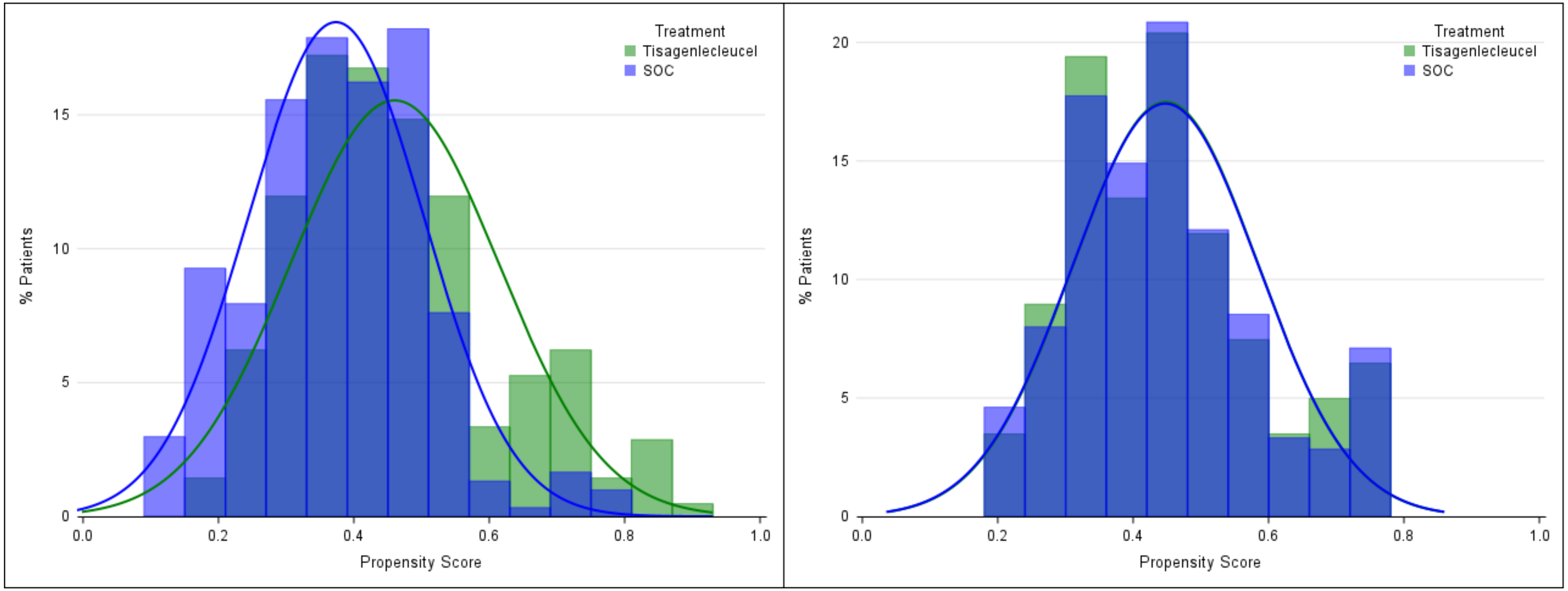

FAS: Full analysis set, PS: Propensity score, SOC: Standard of care

Supplementary Figure 2: Histogram and density plot, ITT, unweighted (left) and weighted (right)

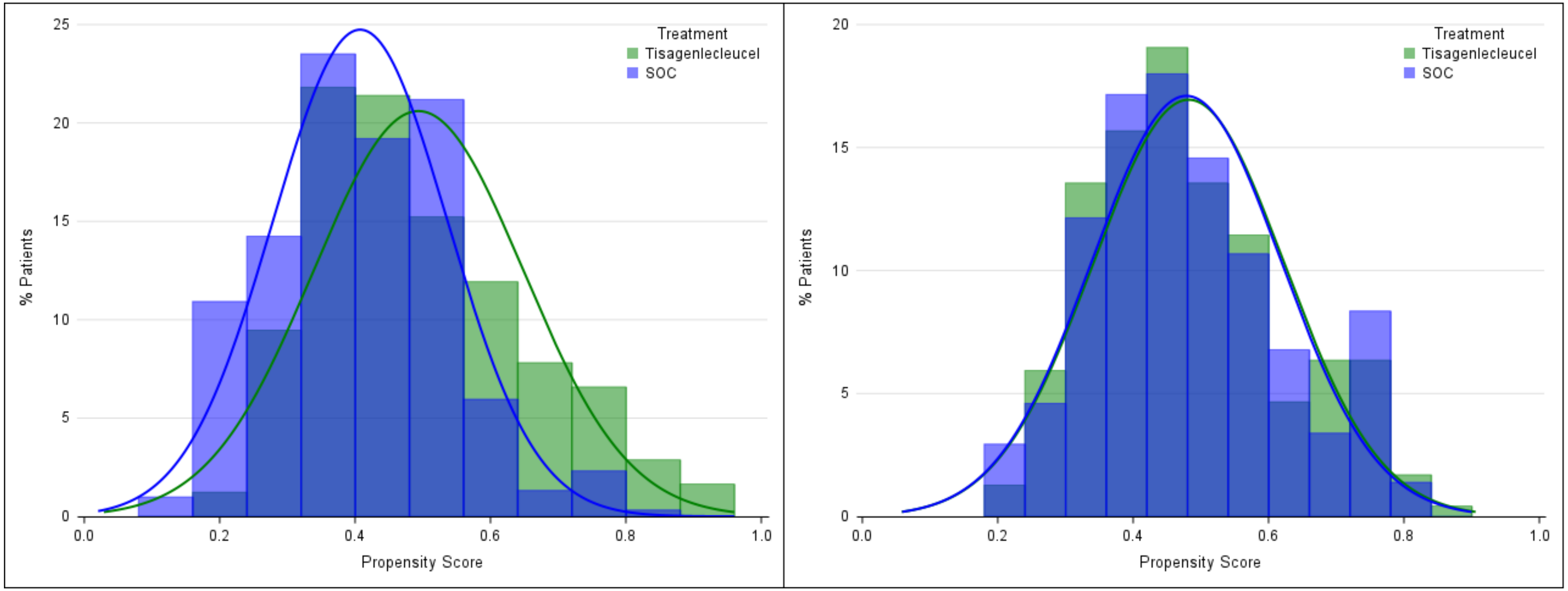

ITT: Intention to treat, PS: Propensity score, SOC: Standard of care

Supplementary Figure 3: Standardized mean differences, balance plot, unweighted and weighted, FAS

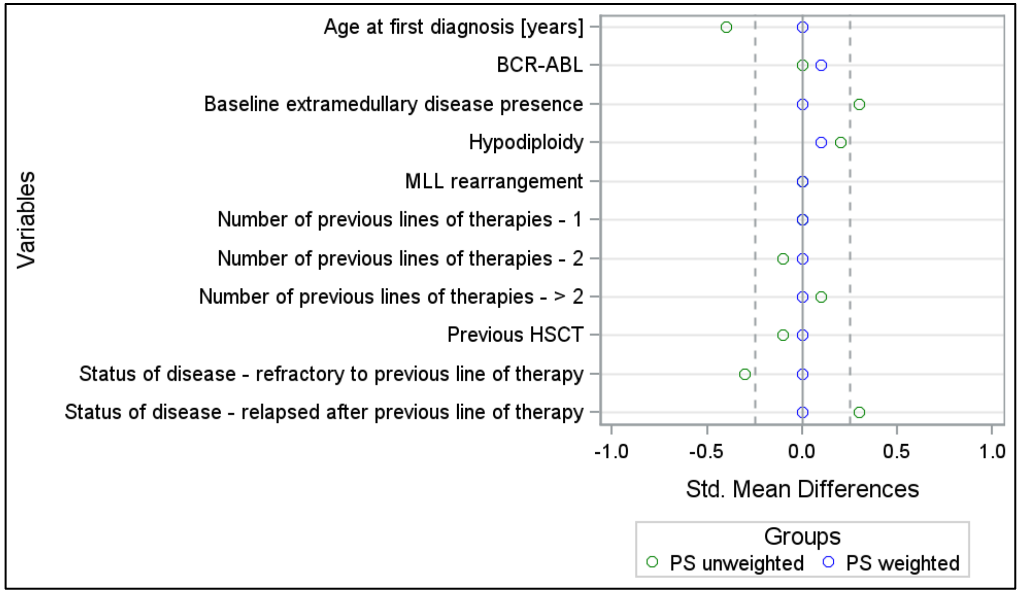

FAS: Full analysis set, HSCT: Hematopoietic stem cell transplantation, MLL: Mixed-lineage leukemia, PS: Propensity Score

Note: Using a threshold of ISMDI ≤ 0.25 to identify imbalance in the calculated standardized mean differences

Supplementary Figure 4: Standardized mean differences, balance plot, unweighted and weighted, ITT

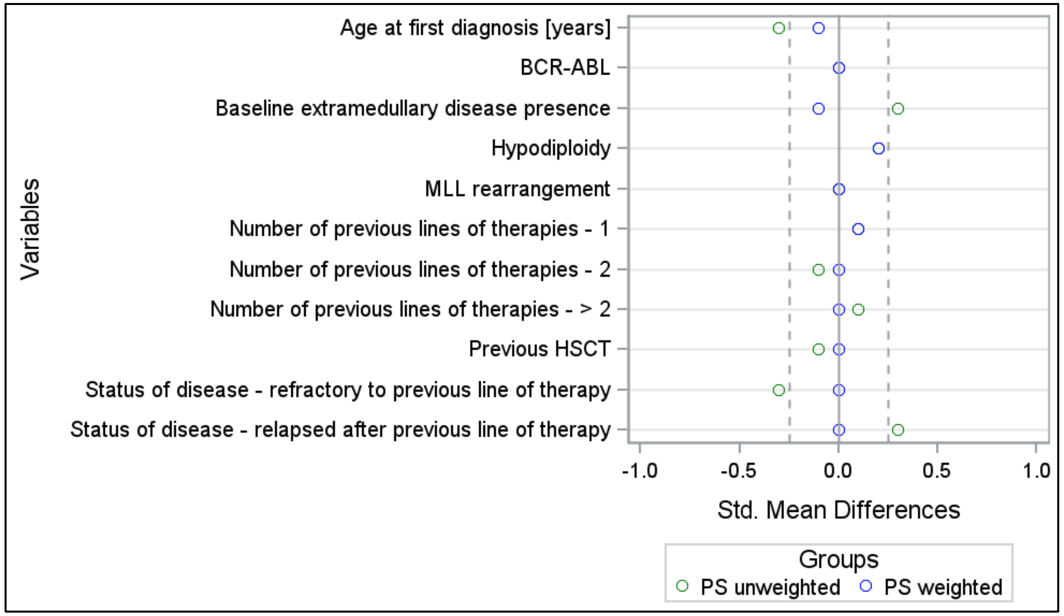

HSCT: Hematopoietic stem cell transplantation, ITT: Intention to treat, MLL: Mixed lineage leukemia, PS: Propensity Score

Note: Using a threshold of ISMDI ≤ 0.25 to identify imbalance in the calculated standardized mean differences
